# Effect of Locally-Administered Pioglitazone on the Survival of Dermal Skin Flaps in an Experimental Study on Rats

**DOI:** 10.7759/cureus.108131

**Published:** 2026-05-02

**Authors:** Efthymios D Basagiannis, Christos Damaskos, Nikolaos Garmpis, Stylianos Kykalos, Alexandros Papalampros, Nikolaos Nikiteas

**Affiliations:** 1 Department of Plastic and Reconstructive Surgery, Aleris Specialistkliniken, Stockholm, SWE; 2 Department of Emergency Surgery, Laiko General Hospital, Athens, GRC; 3 Department of General Surgery, N.S. Christeas Laboratory of Experimental Surgery and Surgical Research, Athens, GRC; 4 Department of Propedeutic Surgery, Laiko General Hospital, Athens, GRC; 5 Department of Experimental Surgery, N.S. Christeas Laboratory of Experimental Surgery and Surgical Research, Athens, GRC; 6 Department of Surgery, Laiko General Hospital, Athens, GRC

**Keywords:** flap necrosis, flap survival, ischemia-reperfusion, local pioglitazone, local pioglitazone administration, pparγ agonist, rat model, skin flaps

## Abstract

Background

Distal necrosis remains a common limitation of random-pattern skin flaps due to progressive decline in perfusion and ischemia-reperfusion injury. Pioglitazone, a peroxisome proliferator-activated receptor gamma (PPARγ) agonist, has anti-inflammatory effects, but systemic administration may be limited by adverse effects. This study evaluated whether local pioglitazone delivery improves the survival of a random-pattern dorsal skin flap in rats.

Materials and methods

Sixteen female Wistar rats were allocated into two groups (n=8/group). A cranially based random-pattern dorsal skin flap was elevated in all animals. In the treatment group, pioglitazone was injected subcutaneously into the flap immediately after elevation; the control group received no additional intervention. Flaps were monitored daily, and animals were euthanised on postoperative day eight. Distal flap tissue was harvested, fixed in 10% formalin, processed, and evaluated by Hematoxylin and Eosin (H&E) staining. Necrosis was quantified histologically. Normality was assessed using the Shapiro-Wilk test, and group comparisons were performed using an independent-samples t-test. Severity categories were compared using Chi-Square and Fisher's exact test.

Results

Necrosis values followed normal distribution (Shapiro-Wilk *p*=0.106). Group variances did not differ significantly (Levene F =2.207, *p*=0.160). Mean necrosis was higher in controls (0.825 ± 0.065) than in pioglitazone-treated flaps (0.706 ± 0.102), with a statistically significant between-group difference (t=2.780, *p*=0.015, 95% CI 0.027-0.210). Categorically, controls exhibited predominantly severe necrosis (seven out of eight) whereas pioglitazone-treated flaps were predominantly moderate (six out of eight), necrosis category differed significantly by group (Pearson χ² = 6.349, df = 1, *p*=0.012; Fisher *p*=0.041).

Conclusions

Local subcutaneous pioglitazone administration significantly reduced distal histological necrosis and shifted outcomes toward less severe injury in a stringent random-pattern dorsal flap model, supporting further investigation of localised pioglitazone as an adjunct to improve flap viability while minimising systemic exposure.

## Introduction

Skin defects are a frequent challenge in Plastic and Reconstructive Surgery and commonly arise following trauma, oncologic resection, infection, or chronic wounds. Small defects may be managed by secondary intention; however, a substantial proportion require operative reconstruction in order to restore durable coverage and acceptable functional and aesthetic outcome [1]. Contemporary decision-making is often described by the reconstructive ladder, which conceptually classifies reconstructive options from simpler to more complex approaches, while subsequent refinements such as the reconstructive elevator emphasize selection of the most appropriate technique for the defect rather than strict stepwise escalation [2].

Among available options, local skin flaps remain a first-line option for defect coverage. A flap is defined as a segment of tissue surgically raised and transferred from its native site while maintaining its original blood supply. In the case of cutaneous flaps, the transferred unit typically includes epidermis, dermis, and subcutaneous fat, and may also incorporate fascia in fasciocutaneous variants. The principal advantage of local flaps is replacement with adjacent tissue of similar characteristics such as colour, thickness, consistency and hair density, often providing superior aesthetic integration while avoiding the need for foreign materials (tissue expanders or dermal matrices) and minimising morbidity at distant donor sites [3]. Local flaps are commonly categorised by the mode of transfer (advancement [4], rotation [5], and transposition [6]) and the blood supply. Axial flaps incorporate a named vessel and generally tolerate larger dimensions due to their robust blood supply [7], whereas random-pattern flaps depend on the dermal and subdermal vascular plexuses without a dominant axial artery. For this reason, random-pattern flap dimensions in clinical practice are constrained by length-to-breadth ratios (2:1 or 3:1) to mitigate distal ischemia and necrosis [8].

The perfusion of the skin is supported by interconnected microvascular plexuses distributed across the dermis and subcutis. These plexuses receive blood supply from perforator vessels and communicate between adjacent vascular territories through anastomoses that include choke vessels, which may dilate in response to local ischaemia [9-11]. Cutaneous blood flow is regulated by the interplay of sympathetic vasomotor tone and local factors such as tissue oxygen tension, carbon dioxide accumulation, pH, temperature, and vasoactive mediators released during ischemia [12]. During flap elevation, this balance is disrupted abruptly. Vessel interruption and neural injury initiate ischaemia, anaerobic metabolism, and local acidosis, followed by oxidative stress and inflammatory recruitment. These processes are further amplified after restoration of blood flow through ischaemia-reperfusion injury, which can intensify endothelial dysfunction, microvascular stasis, and distal tissue loss [13,14]. Consequently, partial necrosis remains a common and clinically meaningful limitation of random-pattern flap reconstruction.

Given the persistent clinical burden of partial flap necrosis, multiple strategies have been explored to improve flap viability. Surgical delay remains a well-established method for enhancing perfusion reserve, but it requires a staged procedure and is therefore not always practical in routine reconstructive settings [15]. Among pharmacologic approaches, topical nitroglycerine has shown benefit in reducing flap necrosis, particularly in mastectomy skin flaps, yet its use may be limited by adverse effects such as headache and hypotension and by uncertainty regarding generalisability across flap types and clinical scenarios [16,17]. Hyperbaric oxygen therapy has also been used for compromised grafts and flaps, but its adoption remains constrained by logistical demands, cost, and protocol heterogeneity. Experimental studies have further identified agents such as sildenafil that may improve microvascular perfusion and flap survival, but the breadth of tested compounds and the methodological variability across animal models have made translation difficult. Together, these limitations support the need for adjunctive treatments that are mechanistically relevant, locally deliverable, and potentially easier to integrate into surgical practice.

In this context, pioglitazone is of interest because, beyond its antidiabetic use, it has been associated with improved endothelial function, modulation of oxidative stress and inflammation, and enhanced nitric oxide bioavailability - mechanisms directly relevant to flap ischemia and ischemia-reperfusion injury [18-20]. In a rat random-pattern skin flap model, systemic pharmacologic preconditioning with pioglitazone increased flap survival and this benefit was abolished by nitric oxide synthase inhibition, supporting an NO-dependent protective mechanism [21]. These observations suggest that pioglitazone targets mechanisms central to flap failure, including impaired perfusion, oxidative stress, and inflammation.

However, systemic pioglitazone use is associated with adverse effects such as fluid retention and peripheral oedema, which may limit its attractiveness as a perioperative adjunct [22]. This limitation provides a clear rationale for evaluating local delivery, aiming to preserve microvascular and anti-inflammatory benefits while minimising systemic exposure. Consistent with this concept, locally released pioglitazone from biomaterial systems has been reported to modulate cytokine profiles and support tissue repair in wound-healing models. Therefore, the present experimental study investigates whether locally administered pioglitazone can improve survival of dermal random-pattern skin flaps in rats, with outcomes selected to reflect the established pathophysiology of flap ischemia and ischaemia-reperfusion injury.

## Materials and methods

All experiments were conducted at the N.S Christeas Laboratory of Experimental Surgery and Surgical Research, Medical School, National and Kapodistrian University of Athens, Greece. The study was designed and carried out in accordance with applicable national and European legislation on animal welfare and experimental research. The experimental protocol was approved by the institutional review board and ethical committee (approval no.: 1348419/16-12-2022). Sample size planning was performed prospectively during the original design of the broader experiment using an ANOVA-based framework with α = 0.05 and power (1 - β) = 0.80. The present manuscript reports only the control and pioglitazone groups from that broader experimental design. The final group size was selected with the aim of balancing statistical reliability with ethical minimisation of animal use. Body weight at the time of surgery was 233.8 ± 24.1 g in the control group and 236.3 ± 28.1 g in the pioglitazone group (overall range 202-277 g). 

After the surgical procedure, the animals were housed individually (one animal per cage; 70 × 35 cm) in specially designed laboratory animal rooms under controlled environmental conditions, including a temperature of 20-24°C, relative humidity of 45-65%, and a 12 h light/12 h dark cycle. Cages contained appropriate bedding, nesting material, and environmental enrichment in accordance with current guidelines. Standard laboratory chow and water were provided ad libitum. Postoperatively, animals were maintained under conditions intended to minimise stress, including reduced noise and limited handling, with additional nesting material and thermal support provided when required. Animals were monitored daily throughout the follow-up period for general condition, activity, feeding behavior, wound integrity, and gross flap appearance, including color change and visible demarcation of necrotic tissue. No standardised postoperative scoring sheet was used.

Study design

Animals were allocated into two experimental groups: i) Group A (control, eight rats): elevation and inset of a cranially based random-pattern dorsal skin flap, without additional intervention, ii) Group B (pioglitazone, eight rats): identical flap elevation and inset followed by immediate subcutaneous injection of pioglitazone into the flap.

This study was designed as an exploratory proof-of-concept animal experiment. No formal a priori power calculation was performed. A group size of eight animals per group was selected to provide an initial assessment of treatment effect while limiting animal use in accordance with ethical considerations. For the analysis of necrosis severity categories, contingency tables were constructed. Given the small sample size (n=8 per group) and the likelihood of expected cell counts below five, Fisher’s exact test was used as the primary statistical test to assess the association between treatment group and necrosis severity. Pearson’s Chi-square test was additionally calculated and is reported descriptively for completeness. A p-value <0.05 was considered statistically significant.

Anaesthesia and perioperative preparation

**Figure 1:**
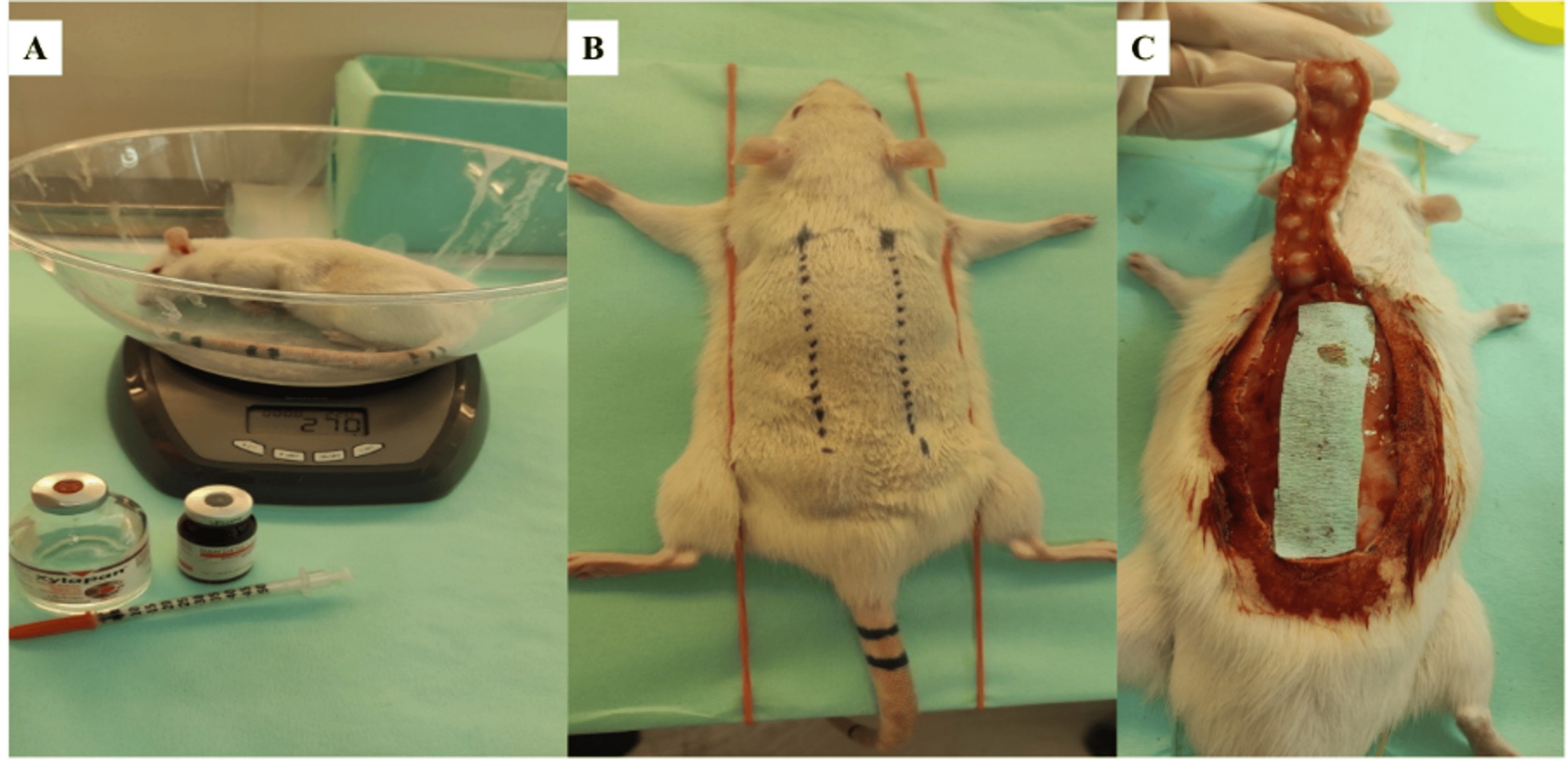
Establishment of the cranially based random-pattern dorsal skin flap model and operative workflow A. Animal weighing and preparation for intraperitoneal anaesthesia (ketamine/xylazine) prior to surgery; B. Preoperative preparation and marking of the planned 8×2 cm cranially based unipedicled dorsal skin flap on the rat dorsum; C. Intraoperative view after flap elevation in the plane superficial to the muscle fascia. A cellulose sheet is placed between the elevated flap and the wound bed to prevent revascularization from the underlying tissues before the flap is returned to its original position and sutured.

General anaesthesia was induced by intraperitoneal administration of ketamine hydrochloride (50 mg/kg) and xylazine hydrochloride (10 mg/kg), providing rapid induction and maintenance of anaesthesia throughout the procedure. Rats were weighed prior to anaesthesia and transferred to the operating room (Figure 1A).

The dorsum was shaved, prepared with povidone-iodine (Betadine), and draped in a sterile surgical field.

Random-pattern dorsal flap model and surgical procedure

A cranially based unipedicled random-pattern dorsal skin flap measuring 8×2 cm (length-to-width ratio 4:1) was designed on the back of each rat (Figure 1B). This ratio exceeds commonly recommended clinical random-flap proportions (e.g., 2:1 or 3:1) and was selected to challenge distal perfusion and enable robust assessment of ischemia-driven necrosis under severe conditions. The flap was incised and elevated using scalpel and scissors in a plane immediately superficial to the muscle fascia, without inclusion of the fascia (Figure 1C).

Following elevation, a biocellulose sheet (Conkote® Biocellulose Dressing, REF 253001, Guangdong, China) was interposed between the flap and the wound bed to impede revascularisation from the recipient bed. The sheet was trimmed to 8 × 2 cm to match the dimensions of the flap and was placed directly beneath the flap without suture fixation. It remained in place throughout the postoperative period and was removed only at the time of euthanasia and specimen harvesting. The flap was then returned to its original anatomical position and sutured with 3-0 Vicryl (Ethicon, Johnson & Johnson, Cincinnati, Ohio, US).

Pioglitazone preparation and administration

Pioglitazone was used in powder form and diluted in sterile normal saline to obtain a 1% (w/v) solution. In the pioglitazone group, 1.0 mL of the pioglitazone solution was administered as 10 subcutaneous injections of 0.1 mL each, distributed uniformly across the flap from the proximal to the distal portion, immediately after flap elevation and prior to final inset.

Postoperative monitoring and humane endpoints

Flaps were monitored on a daily basis for a total of eight days. General condition, wound integrity, and flap appearance were assessed at each time point. No animal died or developed severe complications during the experimental procedure.

Euthanasia and specimen harvesting

On postoperative day eight, tissue samples were obtained from the distal portion of each skin flap, corresponding to the region most susceptible to ischemic injury. Specimens were fixed in 10% neutral buffered formalin, processed routinely, embedded in paraffin, and sectioned at 3 μm thickness. Sections were stained with hematoxylin and eosin (H&E) fixed in paraffin blocks, dehydrated and embedded (TissueTek6, Sakura Finetek Japan Co., Ltd., Tokyo, Japan) and examined by light microscopy. Histological assessment focused on ischaemic-necrotic changes involving the epidermis, dermis, and subcutaneous tissue. Necrosis was quantified as the proportion of the examined distal flap section showing histological necrotic change.

## Results

Histological assessment and quantification of necrosis

**Figure 2:**
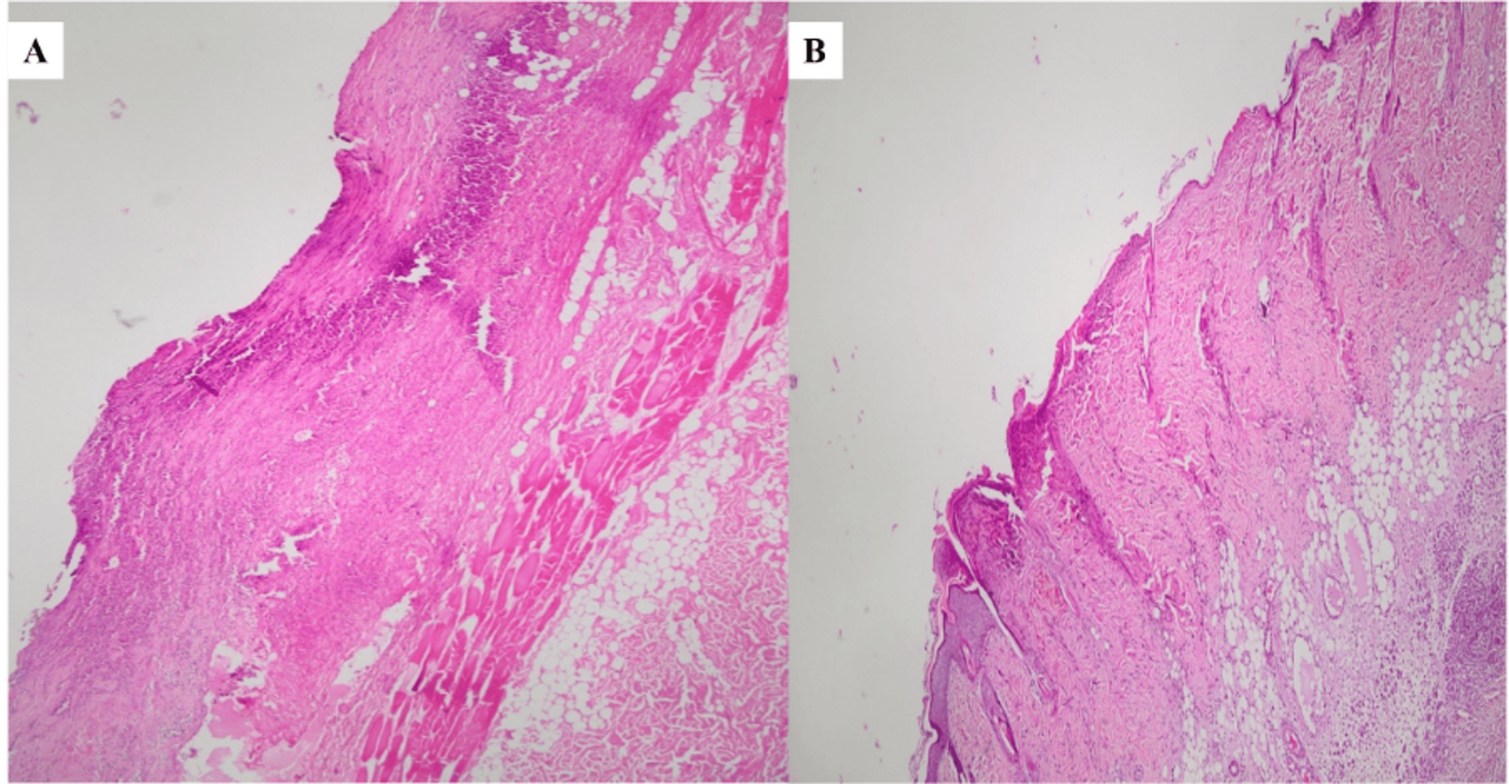
Representative histology of distal flap tissue using H&E staining, demonstrating reduced necrosis following local pioglitazone treatment. According to the hematoxylin-eosin (H&E) staining method (x100 magnification), the blue or purple hematoxylin stains cell nuclei, ribosomes and rough endoplasmic reticulum (basophilic structures) while the pink or red eosin stains cytoplasm and extracellular matrix (acidophilic structures). This dual staining creates high contrast, showing blue nuclei against pink backgrounds, revealing tissue architecture and cellular morphology. A. Group A showing extensive necrotic changes in the distal flap, with loss of normal architecture across the epidermis, dermis, and subcutaneous tissue; B. Group B showing comparatively better preservation of tissue structure and a lower extent of necrotic alteration in the distal flap.

Observation under the optic microscope revealed necrotic alterations of all skin layers and allowed for quantitative evaluation (Figure 2).

More specifically, the percentage of necrosis of the various layers was evaluated and quantified. In both groups, the distal flap demonstrated ischaemia-related tissue injury, including necrotic alterations involving the epidermis, dermis, and subcutaneous tissue. Necrosis was consistently more extensive in group A with a mean necrosis proportion of 0.825 ± 0.065 (range 0.70-0.90) (Figure 2A, Table 1), whereas group B exhibited preservation of tissue architecture and a lower degree of necrotic change, indicating improved distal flap survival with a mean proportion of 0.706 ± 0.102 (range 0.60-0.85) (Figure 2B, Table 1).

**Table 1:** Quantitative comparison of necrosis between the study groups

Rat	Group	Necrosis proportion
1	A	0.80
2		0.85
3		0.70
4		0.90
5		0.80
6		0.80
7		0.85
8		0.90
9	B	0.85
10		0.75
11		0.65
12		0.65
13		0.85
14		0.70
15		0.60
16		0.60

Statistical analysis: control of normal distribution

Prior to inferential testing, the distribution of the necrosis variable was examined. Given a total sample size of 16 animals, normality was tested using the Shapiro-Wilk procedure. Necrosis values were consistent with a normal distribution (*p*=0.106). Descriptive statistics for necrosis across all animals are shown in Table 2 (mean 0.76, median 0.80, standard deviation 0.10, Q1 0.66, Q2 0.80, Q3 0.85).

**Table 2:** Descriptive statistics of necrosis (all animals, n=16) SD: Standard deviation; Q: Quartile.

Descriptives	Necrosis proportion
Mean	0.76
Median	0.80
SD	0.10
Q1	0.66
Q2	0.80
Q3	0.85

**Figure 3:**
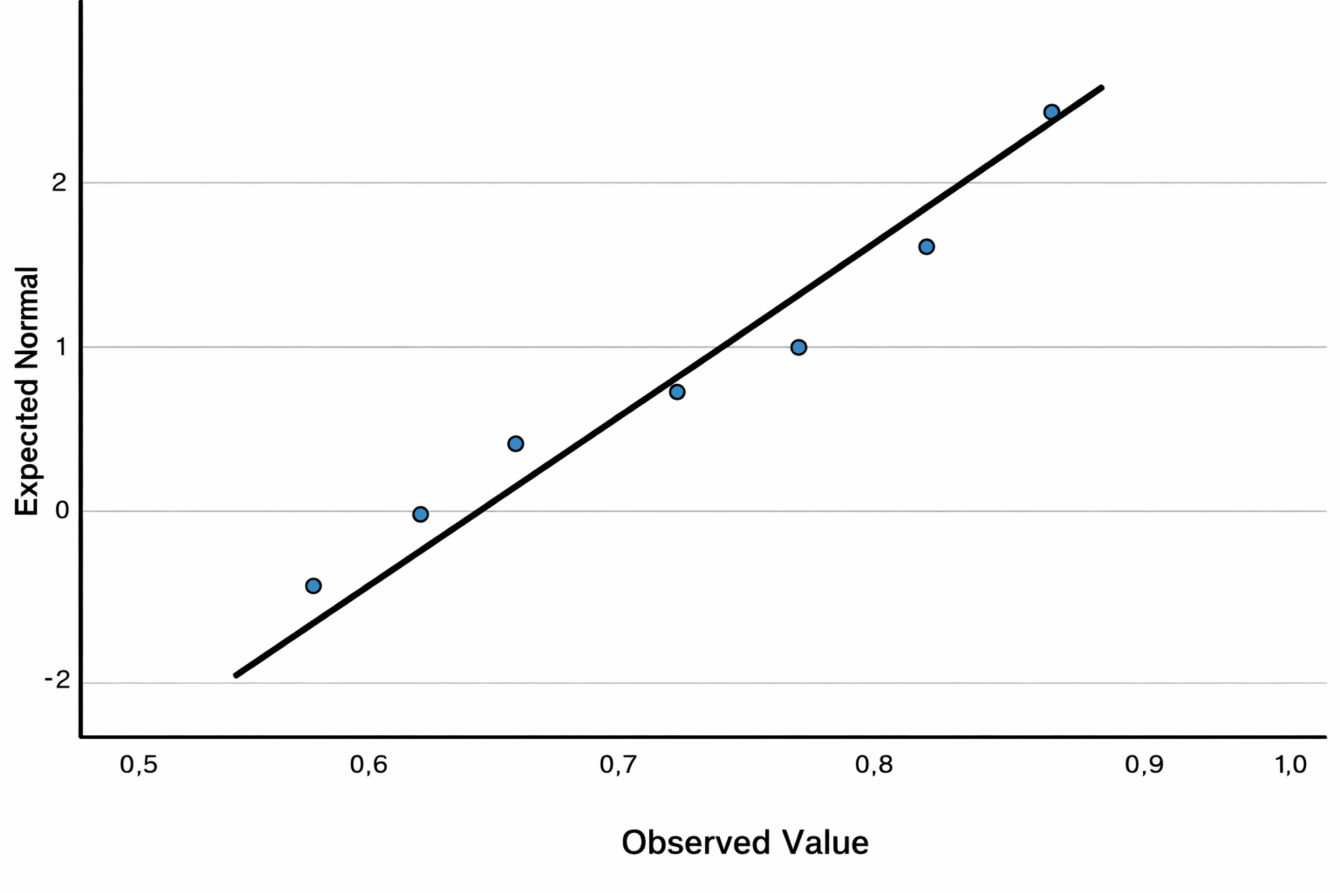
Normality plot (Q-Q plot) for the necrosis variable (n=16)

The corresponding normality plot is provided in Figure 3. 

Visual inspection of the Q-Q plot showed no major deviation from the reference line, supporting the assumption of approximate normality.

Statistical analysis: independent t-test

To evaluate whether necrosis differed between groups, an independent-samples t-test was performed. The null hypothesis was defined as: H0: there is no statistically significant difference between the two groups as to their degree of necrosis. Group-level central values and dispersion are presented in Table 3.

**Table 3:** Descriptive statistics of necrosis by group SD: Standard deviation; SE: Standard error; CI: Confidence interval.

Group	Necrosis						
	Mean	SD	SE	Lower 95% CI for the mean	Upper 95% CI for the mean	Minimum	Maximum
A	0.825	0.0654	0.0231	0.77	0.88	0.70	0.90
B	0.706	0.1015	0.0359	0.62	0.79	0.60	0.85

Group A showed a higher necrosis value (0.825) with standard deviation 0.0654, while Group B showed a lower necrosis value (0.7062) with standard deviation 0.10155. Both groups included an equal number of observations (n=8), supporting a balanced comparison.

To verify the assumption of equal variances, Levene’s test was applied and yielded F=2.207 with *p*=0.160, indicating no statistically significant difference in variances between groups. The independent t-test demonstrated a statistically significant group difference in necrosis (t=2.780, *p*=0.015). The 95% confidence interval for the between-group difference ranged from 0.02713 to 0.21037. Since 0 was not included in the confidence interval, the observed difference is unlikely to be due to random variation alone. The reported difference between the central values was positive (0.11875), supporting greater necrosis in group A relative to group B (Table 3).

Necrosis crosstabulation: definition of categories

For descriptive purposes only, the necrosis proportion was secondarily grouped into author-defined categories of mild (≤0.50), moderate (>0.50 to ≤0.75), and severe (>0.75). These thresholds were not based on a validated histopathologic classification system and should be interpreted as an exploratory descriptive framework only. Under this classification, both groups contained cases of moderate and severe necrosis.

Necrosis crosstabulation: distribution and association testing

**Figure 4:**
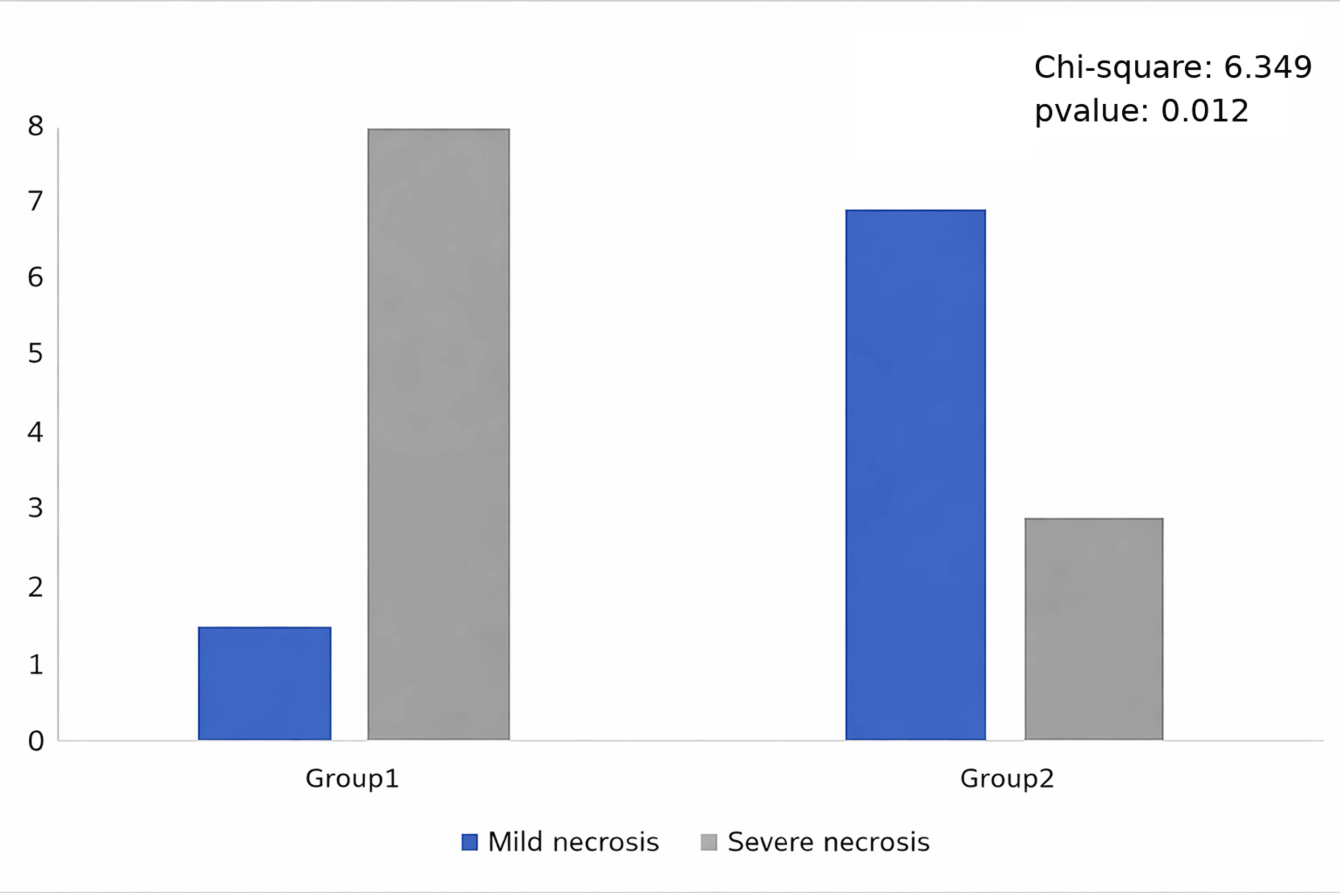
Distribution of necrosis category by group Bar chart showing the number of animals classified as moderate versus severe necrosis in Group A and Group B.

The distribution of necrosis categories by group is presented in Table 4 and illustrated in Figure 4.

**Table 4:** Distribution of necrosis category by group Bar chart showing the number of animals classified as moderate and severe necrosis in each group. Group A (control) demonstrates a predominance of severe necrosis (7/8, 87.5%), whereas Group B (pioglitazone-treated) shows a predominance of moderate necrosis (6/8, 75%), indicating a shift toward less severe tissue injury following local pioglitazone administration.

Group	Necrosis		
	Mild	Moderate	Severe
A (Control)	0 (0)%	1 (12.5%)	7 (87.5%)
B (Pioglitazone)	0 (0)%	6 (75%)	2 (25%)

In Group A, seven out of eight animals (87.5%) were classified as severe and one out of eight (12.5%) as moderate necrosis. In Group B, two out of eight animals (25%) were classified as severe and six out of eight (75%) as moderate necrosis.

To assess the association between treatment group and necrosis severity, Fisher’s exact test was applied as the primary analysis, demonstrating a statistically significant association (*p*=0.041). This finding indicates that necrosis severity differs significantly between groups, with pioglitazone-treated animals showing a shift toward less severe outcomes.

Pearson’s Chi-square test yielded a similar result (χ² = 6.349, df=1, *p*=0.012) and is reported as a supplementary analysis.

## Discussion

The present experimental study evaluated whether local subcutaneous administration of pioglitazone immediately after elevation of a cranially based random-pattern dorsal skin flap can improve flap outcome under conditions of marked ischaemic stress. Using histological assessment of the distal flap portion on postoperative day eight, the data indicate that pioglitazone-treated flaps exhibit a significantly lower degree of necrosis and a shift toward less severe injury compared with controls. These findings support the concept that locally delivered pioglitazone can enhance distal flap viability in a model in which survival is intentionally limited by flap geometry and by separation from the wound bed.

Random-pattern flaps are physiologically constrained by their dependence on the dermal and subdermal vascular plexuses rather than a dominant axial vessel. Consequently, perfusion decreases progressively toward the distal end and distal necrosis often represents the limiting complication. The current flap design (8 × 2 cm, 4:1) is more demanding than commonly recommended clinical ratios and was selected to amplify ischaemia and expose treatment effects. In addition, the placement of a cellulose barrier beneath the flap likely minimised inosculation and revascularization from the wound bed, further concentrating dependence on the cranial pedicle. Within this context, the distal histological sampling strategy is appropriate because it targets the region most susceptible to hypoperfusion and ischaemia-reperfusion injury. The current flap design (8 × 2 cm, 4:1) was intentionally more demanding than commonly recommended clinical ratios and was selected to amplify ischaemia and increase sensitivity to treatment effects. However, this also means that the model represents a stringent experimental condition rather than a direct approximation of routine clinical flap reconstruction, and the findings should therefore not be interpreted as directly generalisable to standard clinical flaps.

A notable feature of this study is the emphasis on local rather than systemic administration. Systemic pioglitazone is associated with adverse effects (notably fluid retention and oedema) that can limit clinical attractiveness in the perioperative setting. Demonstrating benefit with local delivery supports a more translationally plausible path: localised exposure at the surgical site with reduced systemic burden. Practically, local delivery could be implemented as intraoperative injection, topical formulation, or controlled-release carrier, provided that local tolerability and systemic absorption profiles are favourable.

Beyond mean differences in necrosis, the categorical severity analysis is useful because it approximates how surgeons conceptualise flap compromise (moderate vs severe tissue loss). Beyond the categorical descriptive findings, the main result of the study is the reduction in continuous histological necrosis proportion in the Group B, indicating greater preservation of distal flap tissue under marked ischaemic stress.

This study has several limitations. First, the sample size was relatively small, which may limit the generalisability of the findings. Second, the use of an animal model may not fully replicate human physiological responses in clinical settings. Third, the follow-up period was limited to eight days, and longer-term outcomes were not assessed. Finally, functional and perfusion-based measurements were not included, which could further clarify the underlying mechanisms of the observed effects.

Future studies should evaluate the dose-response relationship of locally administered pioglitazone and incorporate complementary perfusion-based or molecular endpoints to better define the underlying mechanism. Validation in larger cohorts and in less extreme, more clinically representative flap models will also be important to determine the robustness and translational relevance of the observed effect.

## Conclusions

In conclusion, local pioglitazone administration was associated with reduced distal histological necrosis and improved tissue preservation in a severe random-pattern flap model. These findings support the potential of localised pioglitazone delivery as an experimental adjunct for improving flap viability while limiting systemic exposure. In addition, the observed effect is consistent with mechanisms related to improved microvascular perfusion, modulation of inflammatory responses, and reduction of ischaemia-reperfusion injury at the tissue level. The use of local administration may offer a practical advantage by allowing targeted drug delivery directly to the area at risk, potentially enhancing efficacy while minimising systemic adverse effects.

Although the present findings are encouraging, they should be interpreted within the constraints of an experimental animal model and a relatively small sample size. Further studies are warranted to better define optimal dosing strategies, evaluate functional and perfusion-based outcomes, and confirm these effects in larger and more clinically representative models. If validated, this approach may contribute to the development of simple and clinically applicable strategies to enhance flap survival in reconstructive surgery.

## References

[1] Janis JE, Kwon RK, Attinger CE (2011). The new reconstructive ladder: modifications to the traditional model. Plast Reconstr Surg.

[2] Gottlieb LJ, Krieger LM (1994). From the reconstructive ladder to the reconstructive elevator. Plast Reconstr Surg.

[3] Saber AY, Hohman MH, Dreyer MA (2024). Basic flap design. StatPearls [Internet].

[4] Etzkorn JR, Zito PM, Hohman MH, Council M (2024). Advancement flaps. StatPearls [Internet].

[5] Prohaska J, Sequeira Campos MB, Cook C (2024). Rotation flaps. StatPearls [Internet].

[6] Bednarek RS, Sequeira Campos MB, Hohman MH, Ramsey ML (2025). Transposition flaps. StatPearls [Internet].

[7] Mehta A, Goldman JJ (2026). Axial flaps. StatPearls [Internet].

[8] McGregor IA, Morgan G (1973). Axial and random pattern flaps. Br J Plast Surg.

[9] Ho WT (2025). Enhancing anatomical understanding of the dermal microvascular plexus for improved flap outcomes in axillary hyperhidrosis. Ann Plast Surg.

[10] Yousif NJ, Ye Z, Grunert BK, Gosain AK, Matloub HS, Sanger JR (1998). Analysis of the distribution of cutaneous perforators in cutaneous flaps. Plast Reconstr Surg.

[11] Cao Z, Jiao H, Gan C (2025). Choke anastomosis: a key element acting as a shunt converter between adjacent angiosomes. Ann Plast Surg.

[12] Berry CE, Le T, An N (2024). Pharmacological and cell-based treatments to increase local skin flap viability in animal models. J Transl Med.

[13] McLean TN, Smith BA, Morrison EC, Nasjleti CE, Caffesse RG (1995). Vascular changes following mucoperiosteal flap surgery: a fluorescein angiography study in dogs. J Periodontol.

[14] Dorweiler B, Pruefer D, Andrasi TB, Maksan SM, Schmiedt W, Neufang A, Vahl CF (2007). Ischemia-reperfusion injury: pathophysiology and clinical implications. Eur J Trauma Emerg Surg.

[15] Pearl RM (1981). A unifying theory of the delay phenomenon-recovery from the hyperadrenergic state. Ann Plast Surg.

[16] Wang P, Gu L, Qin Z, Wang Q, Ma J (2020). Efficacy and safety of topical nitroglycerin in the prevention of mastectomy flap necrosis: a systematic review and meta-analysis. Sci Rep.

[17] Vania R, Pranata R, Irwansyah D, Budiman Budiman (2020). Topical nitroglycerin is associated with a reduced mastectomy skin flap necrosis-systematic review and meta-analysis. J Plast Reconstr Aesthet Surg.

[18] Huang PH, Sata M, Nishimatsu H, Sumi M, Hirata Y, Nagai R (2008). Pioglitazone ameliorates endothelial dysfunction and restores ischemia-induced angiogenesis in diabetic mice. Biomed Pharmacother.

[19] Takatori S, Zamami Y, Yabumae N, Hanafusa N, Mio M, Egawa T, Kawasaki H (2008). Pioglitazone opposes neurogenic vascular dysfunction associated with chronic hyperinsulinaemia. Br J Pharmacol.

[20] Zhao Y, Lützen U, Gohlke P, Jiang P, Herdegen T, Culman J (2021). Neuroprotective and antioxidative effects of pioglitazone in brain tissue adjacent to the ischemic core are mediated by PI3K/Akt and Nrf2/ARE pathways. J Mol Med (Berl).

[21] Gillies PS, Dunn CJ (2000). Pioglitazone. Drugs.

[22] Afraz S, Kamran A, Moazzami K, Nezami BG, Dehpour AR (2012). Protective effect of pharmacologic preconditioning with pioglitazone on random-pattern skin flap in rat is mediated by nitric oxide system. J Surg Res.

